# Supplementary material for: Population Assessments of Federally Threatened Everglades Bully in Big Cypress National Preserve, Florida, USA, Using Habitat Suitability Modeling and Micromorphology
Source: Plants (Basel). 2023 Mar 23;12(7):1430. doi: 10.3390/plants12071430 (PMC10097282; doi:10.3390/plants12071430)
Supplement: Supplementary file 1 [file plants-12-01430-s001.zip › plants-2216812-supplementary.pdf]

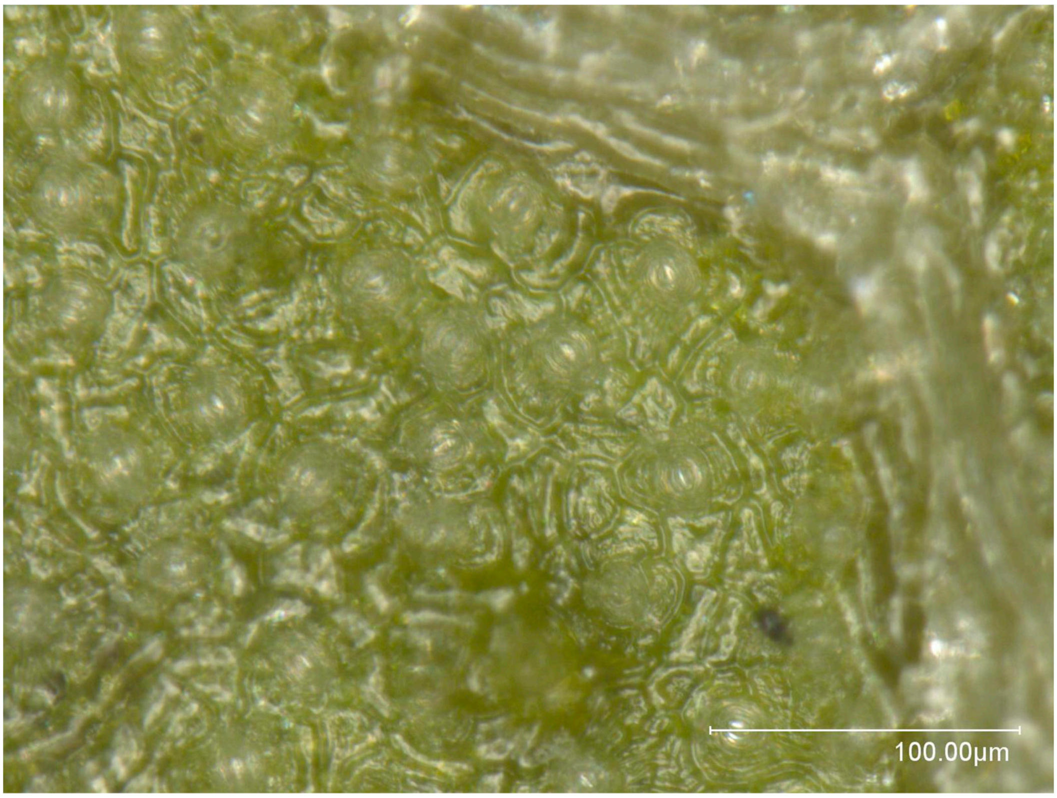

(**a**)

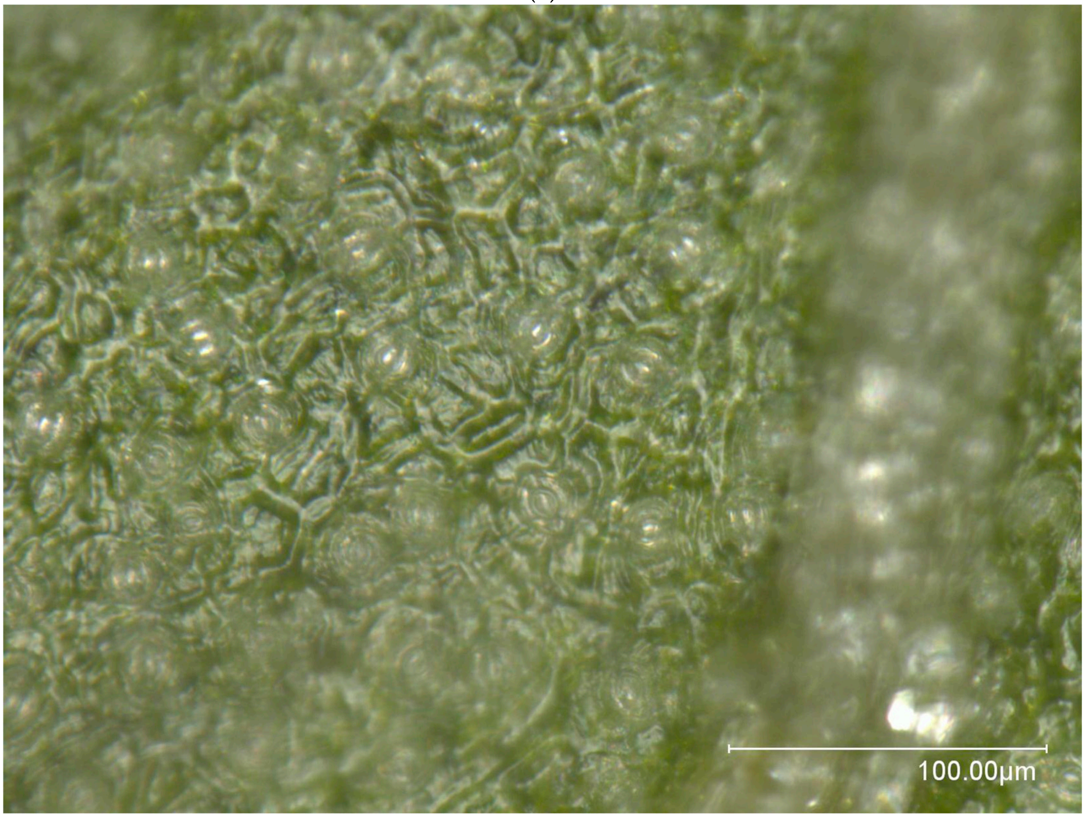

(**b**)

Figure S1. Comparative digital microscope imagery of abaxial laminar surfaces of separate specimens of (**a**) *S. reclinatum* subsp. *austrofloridense* and (**b**) *S. reclinatum* subsp. *reclinatum*.
